# Tailoring resistive switching in Pt/SrTiO_3_ junctions by stoichiometry control

**DOI:** 10.1038/srep11079

**Published:** 2015-06-09

**Authors:** Evgeny Mikheev, Jinwoo Hwang, Adam P. Kajdos, Adam J. Hauser, Susanne Stemmer

**Affiliations:** 1 Materials Department, University of California, Santa Barbara, CA 93106-5050, U.S.A

## Abstract

Resistive switching effects in transition metal oxide-based devices offer new opportunities for information storage and computing technologies. Although it is known that resistive switching is a defect-driven phenomenon, the precise mechanisms are still poorly understood owing to the difficulty of systematically controlling specific point defects. As a result, obtaining reliable and reproducible devices remains a major challenge for this technology. Here, we demonstrate control of resistive switching based on *intentional* manipulation of native point defects. Oxide molecular beam epitaxy is used to systematically investigate the effect of Ti/Sr stoichiometry on resistive switching in high-quality Pt/SrTiO_3_ junctions. We demonstrate resistive switching with improved state retention through the introduction of Ti- and Sr-excess into the near-interface region. More broadly, the results demonstrate the utility of high quality metal/oxide interfaces and explicit control over structural defects to improve control, uniformity, and reproducibility of resistive switching processes. Unintentional interfacial contamination layers, which are present if Schottky contacts are processed at low temperature, can easily dominate the resistive switching characteristics and complicate the interpretation if nonstoichiometry is also present.

Voltage-driven modulation of the electrical resistance of a two-terminal device, commonly referred to as resistive switching, is attractive for next-generation, non-volatile memories, with prospects including very high-density integration and multi-state logic implementation^1^,^2^,^3^,^4^. A current major roadblock for this technology is the lack of reproducibility and uniformity of the resistive switching effect^3^,^5^. This issue is a consequence of the fact that although it is known that resistive switching is caused by point defects^6^,^7^,^8^,^9^,^10^,^11^,^12^,^13^,^14^,^15^,^16^, the specific type(s) of defect(s) that are responsible for the switching process are poorly understood. Different types of defects may exist in typical materials that are used for these devices, making it a challenging phenomenon to control and study.

Metal/Nb:SrTiO_3_ Schottky junctions are a widely investigated materials system for resistive switching^7^,^14^,^15^,^17^,^18^,^19^. They typically exhibit large bipolar switching without the need for an initial forming step. In a previous report^20^, we demonstrated that the emergence of large resistive switching is governed by the quality of the metal/SrTiO_3_ interface. Typical metallization processes use polycrystalline metals that are deposited at room temperature. Such interfaces contain interfacial contamination or defect layers that give rise to large switching effects caused by a voltage-induced modulation of the trapped charge in the layer^20^. The thickness of the interfacial layer determines the voltage drop due to the trapped charge, the degree of Schottky barrier modulation, and thereby the magnitude of the resistive switching effect^20^. In contrast, high-quality, epitaxial Pt(001) contacts processed at high temperature are (nearly) free of such interface layers but they also do not exhibit resistive switching^20^. Because interfacial layers associated with low-quality metallization are unintentional and non-uniform, they are unlikely to yield reproducible device performance. Controlled introduction of defects may offer a route towards reproducible and improved switching devices.

Here, we explore this idea by *intentionally* introducing defects near Pt/Nb:SrTiO_3_ interfaces. This is accomplished by inserting non-stoichiometric, epitaxial SrTiO_3_ interlayers, grown by molecular beam epitaxy (MBE), that contain controlled amounts of Ti- or Sr-excess, respectively. We show that such interlayers dramatically alter the switching behavior and significantly improve state retention, compared to devices that rely on the unintentional interface layers discussed above. Furthermore, the results demonstrate the complexity of behavior that is obtained in the presence of both unintentional defect layers and non-stoichiometry, and the need for high quality materials and interfaces to interpret observed resistive switching phenomena.

A key result from ref. 20 is that it establishes a link between voltage-induced resistance modulation and the voltage drop across an interface layer (*Δ*). The resistance state of the junction is largely determined by the Schottky barrier height (*ϕ*_B_), which is given by the difference between the metal workfunction (*ϕ*_M_) and the electron affinity of SrTiO_3_ (*χ*_STO_). As illustrated in Fig. 1, ϕ_B_ is modified by *Δ*:


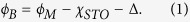


A non-zero *Δ* is a consequence of charge separation between the metal and the doped SrTiO_3_ and its contribution to *Δ* is determined by the interface capacitance. The latter is given by the interface layer thickness (*δ*) and permittivity (*ε*_i_). A fundamental contribution is that of the space charge in the depletion width of SrTiO_3_ (*W*_D_). Any trapped charge present in the system will also contribute. The contribution of a potentially very complex charge profile can be simplified by considering an equivalent charge centroid (*Q*_T_), so that *Δ* can be written as:


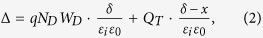


where *x* is the position of *Q*_T_ (as defined in Fig. 1), *N*_D_ the donor doping level in SrTiO_3_, *q* the elementary charge, and *ε*_0_ is the vacuum permittivity. This framework was successful in quantifying resistive switching in terms of voltage-induced modulation of trapped charge (*ΔQ*_T_), which is translated into a modulation of the Schottky barrier height (*Δϕ*_B_). Considering only first order effects, Eqs. (1) and (2) give a useful estimate for the magnitude of the resistive switching effect:


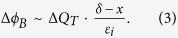


The important conclusion for practical devices is the direct dependence of *Δϕ*_B_ on the interface capacitance (*ε*_i_/*δ*). In the case of typical metals deposited at ambient temperatures to form a resistive switching device, *ε*_i_/*δ* is dominated by uncontrolled contributions from interface contamination and growth-induced disorder^20^. This leads to a statistical spread in *ε*_i_/*δ* values across different samples and devices, which is directly translated into *Δϕ*_B_ and the resistive switching performance, negatively affecting device reproducibility.

A key objective of the work presented here was to achieve improved control over the quantities entering Eq. (3). This involves minimizing unintentional contributions to *ε*_i_/*δ* by using high quality epitaxial Pt contacts and substituting MBE-grown thin films as dominant interface layers. To intentionally define the amount of trapped charge (and thus *ΔQ*_T_), we intentionally introduce defects into the near-interface region. Specifically, we investigate A-site off-stoichiometric SrTiO_3_ as an interface layer. This choice was motivated by the ability to grow epitaxial SrTiO_3_ layers with a well-studied defect chemistry^21^,^22^,^23^,^24^,^25^ on doped SrTiO_3_ substrates, combined with high-quality epitaxial Pt^26^. Previous studies on non-stoichiometric SrTiO_3_ involved polycrystalline films^27^ and metals^28^,^29^. This makes them susceptible to a range of defect-related extrinsic effects, but these studies clearly suggested that resistive switching is sensitive to Ti/Sr stoichiometry.

## Results

### Structural characterization

SrTiO_3_ films were grown on Nb:SrTiO_3_ (001) substrates by hybrid oxide MBE^30^ with five different Ti/Sr ratios. All films thicknesses were between 20 and 30 nm, chosen to avoid strain relaxation for cation off-stoichiometric SrTiO_3_ films, which have an expanded lattice parameter^21^,^31^. Film lattice parameters and stoichiometries, as determined by high-resolution x-ray diffraction and Rutherford backscattering spectrometry (RBS), respectively, are shown in Fig. 2. As can be seen from Fig. 2(a,b), films grown with a TTIP/Sr flux ratio of 47.8 lie in the middle of the MBE growth window^31^, within which stoichiometric SrTiO_3_ films are obtained (Ti/Sr = 1) and the film lattice parameter corresponds to that of the substrate, 3.905 Å [TTIP is titanium tetra isopropoxide used to supply Ti]. Stoichiometric films also exhibit a characteristic c(4 × 4) surface reconstruction^32^ in *in situ* reflection high-energy electron diffraction (RHEED), see Fig. 2(b). Ti and Sr-rich films have expanded out-of-plane lattice parameters and RBS indicates that the sample series shown in Fig. 2 spans a wide range of Ti/Sr stoichiometries, between 0.74 and 1.74, see Fig. 2(c). All samples had smooth surfaces, with streaky RHEED patterns and surface roughness <0.2 nm (root mean square values) in atomic force microscopy. Samples were annealed in O_2_ to eliminate any possible contributions from oxygen vacancies. Cross-section, high-angle annular dark-field (HAADF) scanning transmission electron microscopy (STEM) images of highly off-stoichiometric junctions are shown in Fig. 3. All films are crystalline and epitaxial even in case of very large deviations from stoichiometry. In the Ti-rich regime, locally disordered region are apparent as a change of contrast in the images, suggesting the presence of nanoscale amorphous inclusions of titanium oxide that are typical for Ti-excess films^22^,^23^,^24^. In the Sr-rich regime no extended defects are apparent, which is different from observations in other studies of Sr-rich SrTiO_3_ films, which exhibit Ruddlesden-Popper (RP) defects^21^,^22^,^23^. This could be due to the higher growth rate (~150 nm/h) in hybrid oxide MBE, which may kinetically limit the condensation of Sr into SrO planes, or the high volatility of the TTIP source, which may facilitate the accommodation of Sr-excess via the formation of Ti vacancies, which is not possible when it is evaporated as Ti metal (which has a sticking coefficient of ~1, see ref. 33). In either case, this suggests that Sr-excess is accommodated by the formation of (randomly distributed) point defects, most likely Ti vacancies, as Sr interstitials have very high formation energies^25^.

### Current-Voltage Characteristics

Schottky contacts to the SrTiO_3_/Nb:SrTiO_3_(001) samples consisted of 100-nm thick Pt, deposited by two different processes. The first used polycrystalline Pt grown by e-beam evaporation at room temperature, which will be referred to as Pt(PC) from here on. The second used epitaxial, Pt(001) grown by DC sputtering at high temperature, as described in ref. 26. Without SrTiO_3_ interlayers, Pt(PC)/Nb:SrTiO_3_ junctions show a large current-voltage (*I*-*V*) hysteresis due to the presence of an insulating interfacial layer. Both are suppressed in (001)Pt/Nb:SrTiO_3_ junctions^20^. A total of ten Pt/SrTiO_3_ samples were investigated: five different SrTiO_3_ film stoichiometries and the two types of metallization for each.

Figure 4 shows the forward bias *I*-*V* characteristics of the ten samples. The top and bottom panels show the junctions with Pt(PC) and Pt(001) metallizations, respectively. Horizontally, the panels are ordered according to SrTiO_3_ interlayer stoichiometry, going from Sr-rich on the left to Ti-rich on the right.

As discussed in ref. 20 as well as further below, classic thermionic emission theory provides a good description of the *I*-*V* characteristics in most cases:


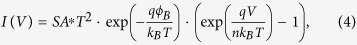


where *S*, A^*^, *k*_B_ and *T* are the junction area, Richardson constant, Boltzmann constant, and temperature, respectively. The fit parameters are the Schottky barrier height *ϕ*_B_ and the ideality factor, *n*. Fits to Eq. (1) are shown are shown as dashed lines in Fig. 4. The values obtained for *ϕ*_B_ and *n* are plotted in Fig. 5 as a function of Ti/Sr stoichiometry in the interlayer.

We first consider the junctions with stoichiometric interlayers (middle panels in both rows). For Pt(PC), a large hysteresis is seen, which is absent for Pt(001). This is similar to the behavior of Pt/Nb:SrTiO_3_ junctions without interlayers, where the resistive switching effect was shown to originate from unintentional interface layers that are present for Pt(PC) but not for Pt(001)^20^. Furthermore, the extracted ideality factors of the Schottky barrier in each case are also similar to those reported in ref. 20. This is consistent with charge carriers from Nb:SrTiO_3_ spreading into the epitaxial SrTiO_3_ interlayers, as expected in the absence of a conduction band offset. As a result, Pt/Nb:SrTiO_3_ and Pt/stoichiometric-SrTiO_3_/Nb:SrTiO_3_ junctions show very similar characteristics.

The *I*-*V* and resistive switching behavior is dramatically altered when the SrTiO_3_ interlayer is non-stoichiometric. When Pt(PC) is used, both Ti- and Sr-rich junctions go through a non-reversible initial step during which strong negative differential resistance (NDR) effects are seen (solid red lines in Fig. 4, the initial resistance state is labeled IRS). After this initial step, junctions can be cycled repeatedly and reproducibly between high and low resistance states (HRS and LRS), see the solid black lines in Fig. 4. This is reminiscent of the “forming” steps necessary to obtain resistive switching effects in many other materials systems.

The values for *ϕ*_B_ and *n* change systematically as a function of Ti/Sr ratio, independent of the type of electrode that is used, as shown in Fig. 5. Also evident is a typical trend for these types of junctions, namely that the Schottky barrier is lowered (and *n* increased) upon switching to LRS. This type of switching, and the effect on the barrier properties, can be attributed to de-trapping of negative charges in the interface layer and/or defects from off-stoichiometry (for quantitative description of this process, see ref. 20).

We note that the effect of the first irreversible step (i.e. the transition from IRS to HRS/LRS) is also described by Eq. (4). This effect is not compatible with an appearance of an Ohmic shunt, i.e. a conductive filament acting as a resistor in parallel with the Schottky barrier, as discussed in refs^34^,^35^. Consequently the physics of the first switching step appear to be similar to the stable switching between HRS and LRS via voltage modulation of trapped charge, except for its permanent nature.

The junctions with Pt(PC) have two contributions in the resistive switching effect, namely the unintentional interface layer discussed in ref. 20, and the defects induced by the non-stoichiometric SrTiO_3_. The junctions with Pt(001) are only controlled by the latter. For Ti-rich interlayers, the use of Pt(001) contacts suppresses the resistive switching effect (see right bottom panels in Fig. 4). Moreover, the conduction mechanism changes from thermionic emission to space-charge limited conduction (SCLC), i.e. the current is bulk-limited instead of interface-limited, yielding a power law dependence of the current on the voltage. This is illustrated by plotting the corresponding data on a log *I* – log *V* scale, as was done in Fig. 4. The suppression of switching by high quality contacts shows that the modification of switching behavior in Ti-rich junctions with Pt(PC) contacts [relative to Pt(PC)/stoichiometric-SrTiO_3_ junctions] originates from an interplay between the defects from the two sources (intentional and unintentional). In contrast, resistive switching in Sr-rich junctions is quite similar for both Pt(001) and Pt(PC) contacts. The magnitude of the effect is reduced for Pt(001), but all four Sr-rich junctions show similar NDR in the first step and *I*-*V* characteristics in HRS and LRS with significant excess current over the standard thermionic emission. The latter can be ascribed to tunneling through the Schottky barrier, resulting in an exponential increase that can also be described by Eq. (1) with a very high *n *~ 7–10, which is indicative of a field emission process^7^,^36^. Again we note that this feature cannot be rationalized as an appearance of a conductive filament acting as a shunt^34^,^35^. Instead this is an interface effect, where the defect states near the metal/oxide interface enable electron tunneling through the Schottky barrier.

### State Retention Characteristics

Figure 6(a) shows the resistance state retention characteristics of devices with Pt(PC) contacts, which are similar to the Pt/SrTiO_3_ junctions with low temperature, polycrystalline Pt reported in the literature. Specifically, the HRS is the stable state, while the LRS is transient and decays back to HRS over time after switching. This decay follows a power law dependence (*I *~ *t*^β^), associated with charge re-trapping in the interface layer^14^,^20^,^37^. The junction with Pt(PC) and Ti/Sr = 1 shows a fast decay with an exponent *β* = 0.35. This value is very consistent with our previous study, which found no appreciable change in LRS decay rate with Pt quality^20^. In contrast, LRS decay rate in both Sr-rich and Ti-rich junctions with Pt(PC) is considerably slower. This is reflected in the decrease of the exponent *β* to ~ 0.1. Most importantly, for Pt(001)-based junctions with Sr-rich SrTiO_3_ interlayers that do show resistive switching effects, both HRS and LRS states are stable with time [Fig. 6(b)]. This corresponds to a very small decay exponent (within the noise, *β *~ 0).

## Discussion

Several conclusions can be drawn from the results shown in Figs 4, 5, 6. First and foremost, the results show that resistive switching with excellent state retention can be achieved by maximizing off-stoichiometry, while concurrently minimizing the unintentional interfacial layer. These conditions are realized for the (001)Pt junctions with Sr-excess SrTiO_3_ interlayers. The observed trends for *β* are consistent with an overlap of two contributions to resistive switching: one is associated with Pt(PC) and decays fast (high *β*); the other one is associated with Ti- or Sr-excess and decays slowly (low *β*). As mentioned above, the most likely point defects that accommodate Sr-excess are Ti-vacancies, which are deep acceptors^25^. These defects appear to play a similar beneficial role, in terms of resistance state retention, as Cr dopants, which are also deep acceptors^9^,^10^. This is consistent with increased time constants associated with defect levels that are deep within the band gap, leading to a stable resistance state.

Secondly, the study shows that junctions that have similar characteristics as many others reported in the literature [Pt(PC) combined with some degree of nonstoichiometry in the bulk] exhibit switching behavior that is complicated by having two origins, namely the unintentional interface layers, and bulk (near interface) defects. Unintentional contamination layers give rise to volatile resistive switching as was already discussed in ref. 20. The results reported here elucidate the nature of the additional contributions that arise from point defects in the bulk. This includes the appearance of an irreversible, forming-like process, which brings the device from an initial resistance state (IRS) to the first LRS. It appears *only in the presence of non-stoichiometry in the SrTiO*_*3*_. The IRS state is independent of the presence of the unintentional contamination layer, as it also appears in the junctions with Pt(001). The systematic trends in *ϕ*_*B*_ and *n* in the IRS as a function of Ti/Sr stoichiometry also establish a close link between the point defects and the IRS state. For all Sr-rich junctions, the IRS is essentially a more extreme version of the HRS (higher *ϕ*_*B*_ and lower *n* for IRS), whereas in Ti-rich samples the initially very high *n* decreases substantially after the forming process.

The logarithmic time dependence that is common to all junctions based on Pt(PC) indicates that the switching mechanism itself is similar, independent of the stoichiometry in the bulk: switching to the LRS corresponds to de-trapping of charges in the unintentional interface layer, the decay of the LRS reflects their re-trapping^20^. The *kinetics* of this process is, however, modified by the Ti/Sr stoichiometry (as reflected in *β*). This points to defect management as a valuable tool for improving state retention performance. This is even more apparent in the junctions with Pt(001) that do not have an unintentional interfacial layer but have a Sr-excess SrTiO_3_ layer. These junctions show essentially no degradation of either resistance state.

A systematic correlation between nonstoichiometry in the interlayer and the junction characteristics is also apparent in the frequency (*f*) dependence of the junction capacitance (*C*) in HRS, shown in Fig. 7(a,b). Except for the expected low frequency deviations in Pt(001)/Ti-rich SrTiO_3_ junctions (due to the high DC leakage seen in the *I*-*V* curves), the capacitive response follows the well-known power law, *C*~*f*^*m-1*^^38^,^39^. Figure 7(c) shows the (1-*m*) as a function to the Ti/Sr ratio. The interpretation of the magnitude of the exponent *m* in the power law is non-trivial, but generally correlated with defects and the associated relaxation times or hoping probabilities^40^,^41^. A helpful framework for its interpretation is the one of random resistor-capacitor networks^42^,^43^, within which the power law exponent *m* and 1*-m* correspond to the fraction of capacitors and resistors, respectively, in the circuit. A simplistic conclusion is to equate the resistor population (1-*m*) with defect concentrations, but the trends in Fig. 7(c) are more intricate. For instance, using Pt(001) consistently increases (1-*m*), which can be interpreted as removal of the capacitor-like unintentional interface layer. It is thus obvious that *m* cannot be used as a straightforward metric for material quality or defect concentrations, as the Ti/Sr = 1 junction with Pt(001) has a fairly high (1-*m*) = 0.0072 despite a nearly ideal *n* of 1.2. The decrease of (1-*m*) with Ti-excess is likely associated with amorphous Ti regions acting in a capacitor-like fashion when Pt(PC) is used. In contrast, Sr-excess consistently increases (1-*m*), consistent with it being accommodated in form of randomly distributed point defects that act in a resistor-like manner within the random network picture. The validity of this trend and the close numerical values for both Pt(PC) and Pt(001) cases indicate that it is dominated by the intentional Sr-excess and not the interface layers. This is consistent with the above conclusions on improving state retention behavior (i.e. slowing charge trapping kinetics) by increasing off-stoichiometry and suppressing unintentional interface layers. While the interpretation of dielectric relaxation with frequency is challenging, it is clearly correlated with the types of defects that are relevant for resistive switching. It is thus a useful metric and guide in improving the design of resistive switching junctions.

Finally, we would like to return to Eq. (3), which estimates the resistive switching effect as Δ*ϕ*_B_ ~ Δ*Q*_T_·*δ*/*ε*_i_. The contribution of this work is that it controls both the Δ*Q*_T_ and *δ*/*ε*_i_ contributions to the resistive switching. The interface capacitance *δ*/*ε*_i_ is set by using Pt(001), minimizing unintentional defect interface layers, and replacing them with MBE-grown SrTiO_3_. The trapped charge *Q*_T_ is defined by introducing point defect traps via intentional Ti/Sr off-stoichiometry. Such “clean” material systems offer a route towards reproducible resistive switching. The approach should be fairly general and applicable to other material systems, as it applies to any Schottky junction in presence of trapped charge. As discussed in ref. 20, in the case of Pt/Nb:SrTiO_3_ junctions, this Schottky barrier height modulation proceeds by standard filling and emptying of trap states, and is inconsistent with ionic electromigration. However, the approach suggested by Eq. (3) should also apply in systems where a trapped charge profile is modified by motion of charged vacancies under an applied voltage.

**Figure 1:**
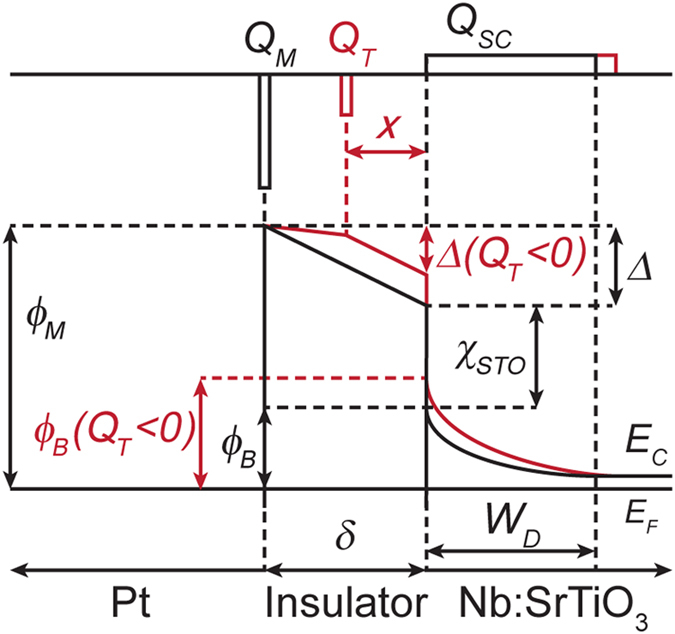
Band structure of a Schottky junction in the presence of an interface layer. The red lines schematically illustrate the effect of a trapped charge centroid *Q*_T_ present within the interface layer.

**Figure 2:**
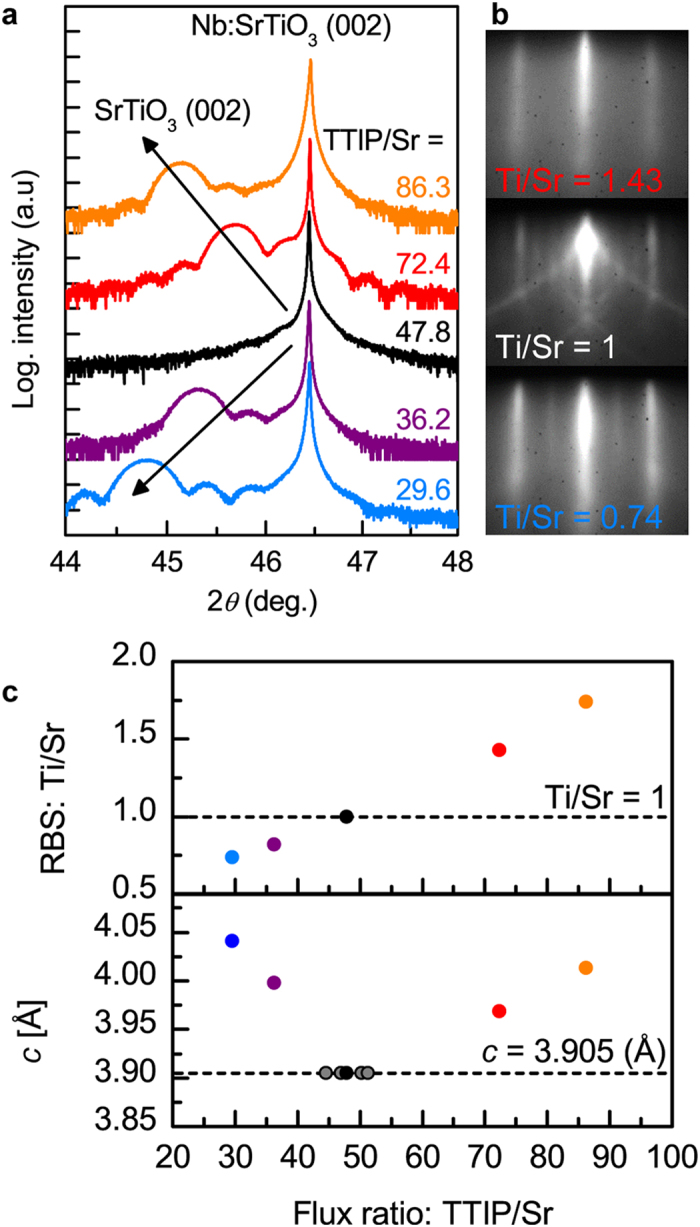
Correlation between lattice parameters, RHEED, and film stoichiometry. **(a)** X-ray diffraction spectra of SrTiO_3_/Nb:SrTiO_3_ films grown with different stoichiometries. The out-of-plane lattice constant increases for TTIP/Sr flux ratios outside of the MBE growth window. **(b)** *In-situ* RHEED patterns along the [110] direction. The streakiness of the patterns indicates smooth film surface and the c(4 × 4) reconstruction for TTIP/Sr = 47.8 is characteristic of stoichiometric growth (Ti/Sr = 1). (**c**) Ti/Sr stoichiometry determined by RBS and correlation with the out-of-plane lattice constant as a function of flux ratio. The grey dots are homoepitaxial calibration growths of SrTiO_3_ on SrTiO_3_ substrates.

**Figure 3:**
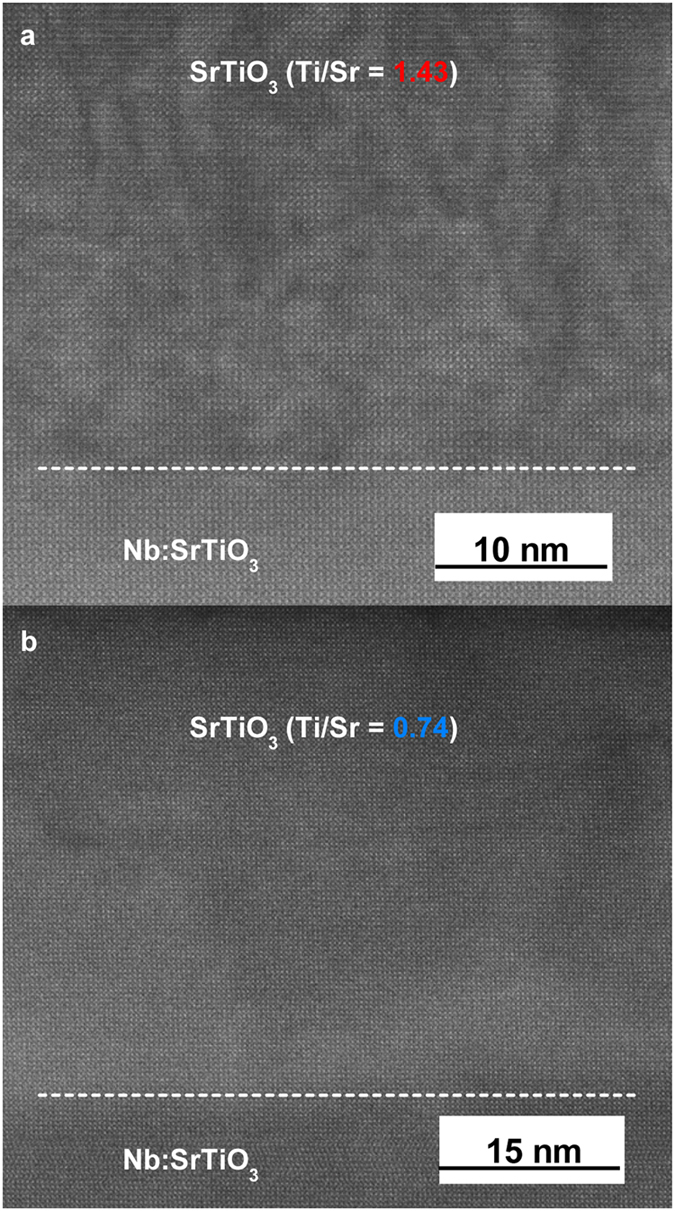
STEM images of nonstoichiometric SrTiO3 films. High-angle, annular dark-field scanning transmission electron microscopy cross-section images for **(a)** Ti-rich and **(b)** Sr-rich SrTiO_3_ films.

**Figure 4:**
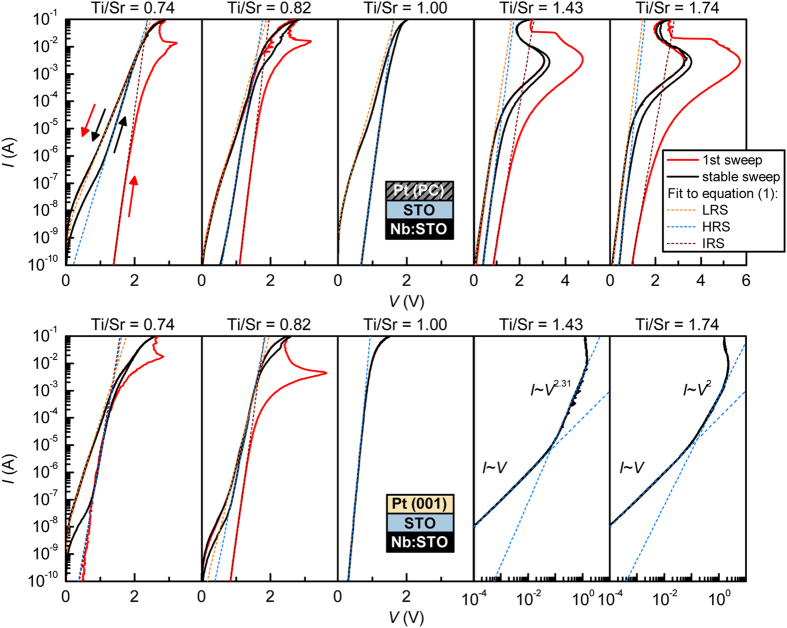
Current-voltage characteristics. Current-voltage characteristics of all Pt/SrTiO_3_/Nb:SrTiO_3_ junctions. Top and bottom rows correspond to junctions with Pt(PC) and Pt(001), respectively. From left to right, the interlayer stoichiometry is tuned from Sr-rich to Ti-rich. If the initial sweep is non-reversible, it is plotted as a red line. Black lines represent stable, reversible sweeps. Dashed lines are thermionic emission fits for IRS, HRS and LRS (initial, high and low resistance states).

**Figure 5:**
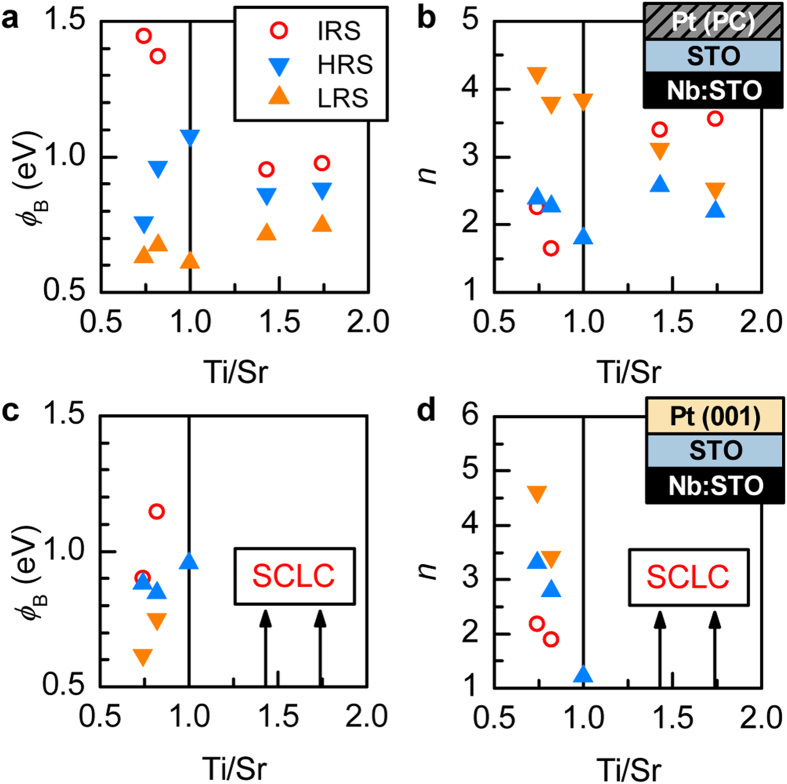
Schottky barrier properties obtained from I-V fits. **(a,b)**: Schottky barrier heights *ϕ*_*B*_ and ideality factors *n* obtained from fitting the data shown in Fig. 3 for junctions with Pt(PC). **(c,d)**: corresponding results for junctions with Pt(001). SCLC indicates the off-stoichiometry range with space-charge limited conduction.

**Figure 6:**
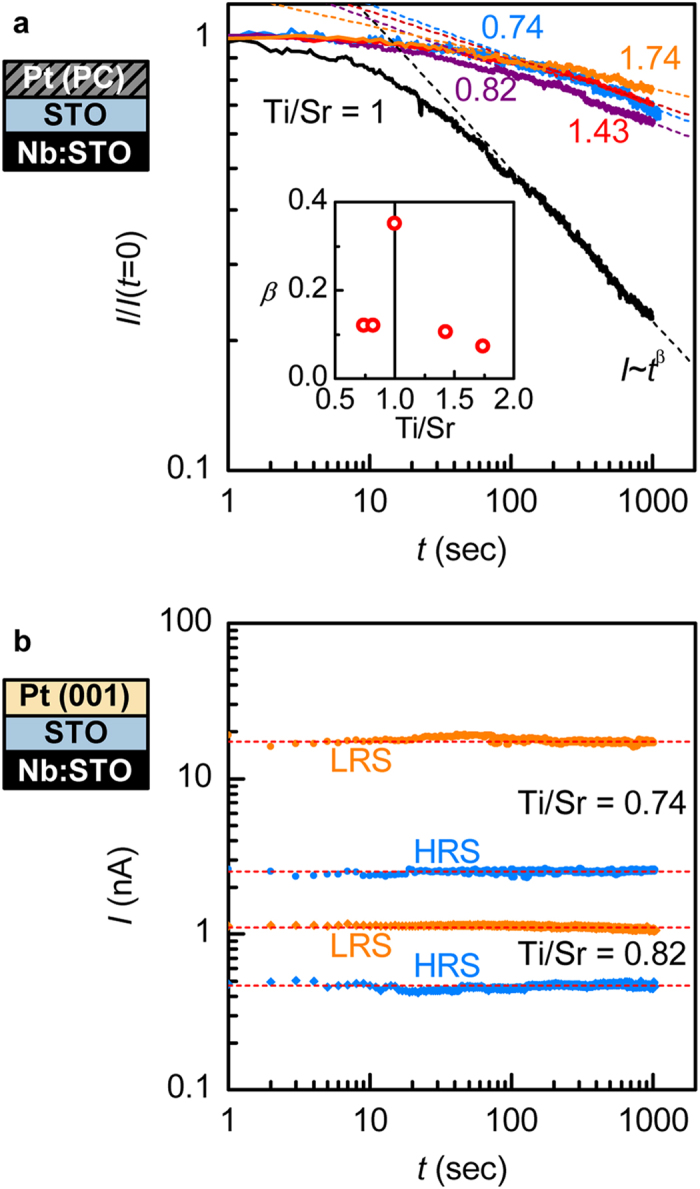
Resistance state retention characteristics. **(a)** Decay of junction current at *V* = 0.1 V for LRS with time after switching from HRS, for junction with Pt(PC). Dashed lines are fits to a power law, with its exponent is shown in the inset. **(b)** State retention for HRS and LRS in Sr-rich junctions with Pt(001).

**Figure 7:**
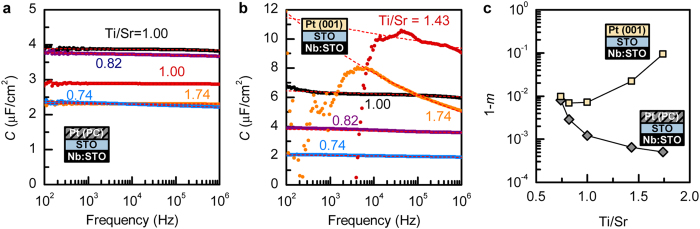
Dielectric relaxation. Frequency-dependence of the junction capacitance in IRS for (**a**) Pt(PC) and (**b**) Pt(001). Frequency-dependence of the junction capacitance in IRS for (**a**) Pt(PC) and (**b**) Pt(001). Dashed lines are fits to *C* ~ *f*^* m-1*^. The exponent (1-*m*) values are shown in **(c)**.

Additionally, Eq. (3) rationalizes the relatively small magnitude of resistive switching observed in the absence of unintentional interface layers, as seen in Figs 4 and 6(b). In this case, Δ*ϕ*_B_ is reduced as the interface layer dielectric constant becomes similar to that of SrTiO_3_, i.e. on the order of *ε*_i_ = 350. Consequently, much higher Δ*ϕ*_B_ could potentially be achieved through improved device design: e.g. substituting the SrTiO_3_ interlayer for perovskites with lower dielectric constants or by intentionally altering the location of trapped charge (i.e. *x* in Fig. 1).

In summary, we have shown that the resistive switching characteristics of oxide/metal junctions are highly sensitive to both (i) an interfacial layer and (ii) point defects in the near interface bulk region, each of which contributes in distinguishable ways to the overall phenomenon. Point defects in the oxide layer (e.g. oxygen vacancies^44^ or deep level dopants like Cr for SrTiO_3_^9^,^10^) are widely accepted to be paramount for resistive switching effects. However, unintentional contamination layers that are typically not removed in low temperature metallization can easily overwhelm other contributions and/or interplay with them in non-obvious ways. Isolating the different contributions requires careful design of the junction and high-quality materials. Here we have demonstrated that non-volatile switching can be obtained by inserting Sr-excess SrTiO_3_ interlayers, while minimizing interface contamination. This makes such junctions an exciting platform for exploring the roles of different defect types in resistive switching effects.

## Methods

### Film growth and characterization

SrTiO_3_ films were grown on Nb:SrTiO_3_(001) substrates by hybrid oxide MBE. Details of the growth procedure and stoichiometry control afforded by this technique are described in detail elsewhere^30^,^31^. The film thicknesses were between 20 and 30 nm. All growths were performed at substrate temperature of 900 °C. Layers with five different Ti/Sr ratios were investigated. Lattice parameter measurements using 2θ-ω high resolution X-ray diffraction (XRD) scans (Philips X’PERT Panalytical MRD Pro Thin-Film Diffractometer) and Rutherford backscattering spectrometry (RBS) were used to determine the film stoichiometry. The stoichiometry of each film was determined by Rutherford backscattering (RBS) measurements carried out at Rutgers University and analyzed using the SIMNRA program.

### Device Fabrication and Measurement

Devices were fabricated by depositing 100-nm thick Pt on stoichiometric or nonstoichiometric SrTiO_3_ films, respectively. Polycrystalline Pt films [Pt(PC)] were grown by e-beam evaporation at room temperature. Epitaxial, Pt(001) was grown by DC sputtering at 825 °C^26^. Square-shaped 45 × 45 μm^2^ top electrodes were patterned by standard photolithography and wet etching of Pt in aqua regia at 60 °C^20^,^45^. A 30-sec anneal in oxygen at 800 °C was performed to eliminate possible contribution from oxygen vacancies. Ohmic contacts to the Nb:SrTiO_3_ substrate consisted of Au(300 nm)/Ni(20 nm)/Al(40 nm) deposited by electron beam evaporation. *I*-*V* characteristics were measured using needle probes and a HP 4155 Semiconductor Parameter Analyzer. Forward bias measurement (switching from HRS or IRS to LRS) were performed in current-controlled mode, ramping from 1 pA to 100 mA and back. Reverse bias sweeps were performed in voltage controlled mode, ramping from zero to a value between -3 and -7 V and back, calibrated to reproducibly revert from LRS to HRS.

## Additional Information

**How to cite this article**: Mikheev, E. *et al*. Tailoring resistive switching in Pt/SrTiO_3_ junctions by stoichiometry control. *Sci. Rep*. **5**, 11079; doi: 10.1038/srep11079 (2015).
